# Hydrocephalus-associated persistent pain: a preclinical investigation correlating brain fluid imbalance and sensory alterations

**DOI:** 10.1186/s12987-026-00838-x

**Published:** 2026-07-07

**Authors:** Clara Ciampi, Samuele Trisolini, Lorenzo Di Cesare Mannelli, Elena Ferri, Carla Ghelardini, Flavio Giordano, Laura Micheli

**Affiliations:** 1 Department of Neurosciences, Psychology, Drug Research and Child Health (NEUROFARBA), Section of Pharmacology and Toxicology, University of Florence, Florence, Italy; 2 Department of Neurosurgery, Meyer Children’s Hospital IRCCS, Florence, Italy

**Keywords:** AQP4, Astrocytes, Microglia, Fluid homeostasis, Hydrocephalus, Persistent pain

## Abstract

**Background:**

Hydrocephalus is a neurological disorder characterized by ventricular enlargement, elevated intracranial pressure and long-term complications, including cognitive dysfunction, motor deficits and pain. Despite the relevance of hydrocephalus-associated pain (characterized by peripheral sensory disturbances and central sensitization) this aspect is neglected both in clinical practice and animal studies.

**Methods:**

We characterized persistent pain in a rat model of kaolin-induced hydrocephalus, investigating behavioural, pharmacological, molecular, and cellular aspects, to improve translational relevance. A particular focus was placed on the role of glial cells in the underlying pathophysiology: astrocytes and astrocytic protein aquaporin-4 (AQP4) and microglia. Analyses were conducted separately in male and female rats as an exploratory approach to examine potential sex-related differences in hydrocephalus-associated pain, an aspect that has been largely overlooked in previous preclinical studies.

**Results:**

Induction of hydrocephalus resulted in a significant reduction in pain threshold, as evidenced by the development of peripheral and cephalic hypersensitivity, and spontaneous pain. This pain phenotype is of mixed origin, encompassing both nociceptive and neuropathic components, and shows limited responsiveness to conventional pain therapy. Histological analysis revealed activation of ependymal cells, likely triggered by increased intraventricular pressure. In brain regions implicated in pain processing, particularly the periaqueductal gray (PAG), astrocytes are altered in terms of quantity, simplified morphology, and diminished AQP4 expression, while microglial cells switched to an activated phenotype, suggesting primary mechanical compression damage leading to cellular distress. In downstream areas, in the dorsal horns of the spinal cord, both astrocytes and microglia displayed overt activation, suggestive of secondary reactive changes at spinal level, in line with the hypothesis of central sensitization.

**Conclusions:**

The regional specificity of these alterations underscores the complexity of hydrocephalus-related pain mechanisms and emphasizes the urgent need for novel therapeutic options to manage pain in hydrocephalus, a clinical challenge that remains inadequately addressed.

**Supplementary Information:**

The online version contains supplementary material available at https://doi.org/10.1186/s12987-026-00838-x.

## Background

Hydrocephalus is a neurological condition characterized by abnormal accumulation of cerebrospinal fluid (CSF) within the ventricular system, leading to ventricular dilation and increased intracranial pressure [1]. The skull is similar to a rigid box and the components within it (namely, blood, CSF, and brain tissue) maintain a volume equilibrium in accordance with the Monro-Kellie law [2]. In presence of hydrocephalus, this equilibrium is disrupted: the brain undergoes deformation and accumulates fluid to buffer pressure changes. Hydrocephalus is thus configured as a disorder of brain and ependymal compliance to arterial systolic wave pulsation: a loss of intracranial “elasticity”. This dysfunction is accompanied by widespread cellular damage in the central nervous system (CNS) [3], and this damage drives the long-term alterations observed even in well-controlled hydrocephalus, including cognitive deficits, motor impairments and pain. Despite the impact and relevance of hydrocephalus-associated pain, relate to peripheral sensory disturbances (including allodynia and hyperalgesia) and central sensitization (CNS becomes hyper-reactive to pain signals), this is an aspect that remains overlooked in clinical guidelines and has not been the object of extensive evaluation in preclinical studies, still representing a grey area in research. In a recently published review [4] pain has been recommended among the ideal endpoint measurements for hydrocephalus, in order to refine animal research aimed at evaluating treatment impact on the disease. Currently, the only available intervention for hydrocephalus is surgery, and there is essentially no effective pharmacotherapy [5]. Therefore, the identification of new non-invasive treatments for hydrocephalus and its associated long-term outcomes represents a research priority. Among the pharmacological options diuretics such as acetazolamide and furosemide are used to reduce CSF production at the level of the choroid plexus, but there is insufficient scientific evidence to prove that acting on this mechanism is an effective strategy [6]. Mannitol, an osmotic agent, is used to “dry” brain parenchyma, but does not drain the ventricles and is not indeed a treatment for hydrocephalus, but rather for intracranial hypertension [5].

Before translationally meaningful results can be achieved, a critical prerequisite is the refinement of animal models to incorporate experimental endpoints that are increasingly specific to the clinical problem of hydrocephalus-associated pain. In a field that remains largely unexplored, such refinement represents not an ancillary goal, but the necessary foundation upon which future translational progress can be built. In this context, the aim of the present study was to investigate in depth for the first time the pain associated with hydrocephalus in a preclinical context, using a widely studied and validated rat model of hydrocephalus, induced by kaolin injection into the cisterna magna. The study examined behavioural, pharmacological, molecular, and cellular aspects, with the goal of identifying potential therapeutic targets for the treatment of hydrocephalus-associated pain. Particular attention was given to the characterization of astrocyte, glial cells essential for both pain modulation and maintenance of hydrodynamic balance, through the presence of astrocytic protein aquaporin-4 (AQP4) [7] and microglia, of particular relevance in the context of central sensitization and sustained neuroinflammation [8, 9].

## Materials and methods

### Experimental design

The primary objective of this research was to investigate the correlation between CSF dynamics impairment and altered pain threshold. We tested the hypothesis that kaolin-induced hydrocephalus in rats leads to modified pain sensitivity, based on clinical observations of pain, peripheral sensory alterations and central centralization in patients with hydrocephalus or other CSF imbalance–related conditions, such as idiopathic intracranial hypertension [4, 10]. Secondary endpoints included group-wise comparisons of ventricular enlargement and histological features of brain tissue damage. Molecular and cellular analyses focused on the expression of key glial markers. Glial fibrillary acid protein (GFAP), AQP4 expression and Ionized calcium-binding adapter molecule 1 (Iba1) were assessed by immunofluorescence in CNS regions implicated in pain modulation and hydrocephalus pathology, including periaqueductal gray, somatosensory cortex, dorsal horns of the spinal cord, lateral ventricles and ependymal lining. AQP1 expression was evaluated in the choroid plexus by immunofluorescence, and transcript levels of GFAP, CAIII, and CAIV were analysed using qPCR.

Experimental animals were 3-week-old Sprague Dawley rats derived from in-house breeding. To minimize litter-related confounding, small groups of pups from each litter were distributed across both control and hydrocephalus groups. No formal randomization software was used.

Pain threshold was evaluated at 1, 3, and 5 weeks (corresponding to animal age in post-natal days (PND) of PND28, PND42 and PND56, respectively) after hydrocephalus induction to capture both acute and chronic stages of hydrocephalus [11]. In this time-course study, the control group of sex- and age-matched healthy animals serves as the reference for physiological comparison at each time point.

Subsequent analyses focused on the 3-week point, when ventriculomegaly is stable [11], and motor function is preserved.

Sample size estimation (G*Power 3.1.9.2) was based on previous studies using similar behavioral assay [12–15] with α = 0.05, power = 0.80, and large effect size (Cohen’s d = 1.6). Target size was 8 animals/group, but actual numbers varied due to breeding constraints, animal exclusions, and technical challenges during tissue collection. Nevertheless, statistically significant differences between control and treated groups were consistently observed.

Adverse events in this model occur at a rate of ~ 7% [16]. Animals with significant health issues (> 20% weight loss, seizures, infection) were excluded (5 out of 92 treated; none of 75 controls).

Although full blinding was not feasible due to overt phenotypes, investigators were blinded to animals’ allocation during behavioural tests, to reduce observational bias.

The numerosity for each individual measurement (behavioural or histological) is reported in the figure legends. Sample size across measurements can vary reflecting sources of attrition that occurred during the experimental sessions: in behavioural assays, individual animals were occasionally excluded when they failed to respond or exceeded the pre-defined cut-off threshold for a given test; in histological analyses, technical difficulties during tissue collection and processing sometimes limited the number of samples for a given target region.

Full description of all animal cohorts involved in the studies are detailed in a time chart in the supplementary material (Supplementary Figure S1).

### Animals

Male and female Sprague Dawley rats born within the CeSAL (Laboratory Animal Housing Centre, University of Florence) from pregnant rats were used. Pregnant rats were supplied by Charles River (Lecco, Italy). After their arrival, pregnant rats were housed at CeSAL in single cages (dimensions 26 × 41 cm), in which they gave birth. Litters of male and female rats were kept inside their respective maternal cages until the weaning age of 21 days. Afterwards, the rats were divided by sex and housed separately, four per cage. All animals were fed a standard laboratory diet and water ad libitum, maintained at 23 ± 1 °C and on a 12 h light/dark cycle, with lights turned on at 7 am. All animal manipulations were carried out in accordance with Directive 2010/63/EU of the European Parliament and of the Council of the European Union (22 September 2010) on the protection of animals used for scientific purposes. The University of Florence’s ethics policy is in accordance with the U.S. National Institutes of Health Guide for the Care and Use of Laboratory Animals (NIH Publication No. 85–23, revised 1996; University Assurance Number of Florence: A5278– 01). Formal approval to conduct the described experiments was obtained from the Italian Ministry of Health (n. 32/2025-PR) and from the Animal Subjects Review Board of the University of Florence. The results are reported according to the ARRIVE guidelines for animal research [17]. All efforts have been made to minimize animal suffering and to reduce the number of animals used.

### In vivo model of hydrocephalus induced by kaolin injection into the cisterna magna

The protocol described by Chen and colleagues has been followed with minimal variations [11]. Upon reaching 3 weeks of age, the rats have been anesthetized by intraperitoneal injection of a mixture of ketamine 60 mg/kg and xylazine 6 mg/kg. The depth of anaesthesia was evaluated and monitored throughout the procedure via toe-pinch test. An ophthalmic ointment was applied at the beginning of the procedures to avoid ocular dryness, repeating the application during the operation if necessary. The animal’s body temperature was monitored constantly via rectal probe and maintained in the 36.5–37 °C range using a heating pad. Rat’s head and neck were shaved and the exposed skin was then sterilized with a swab soaked in 70% ethanol. Animals were placed on a stereotaxic frame (Standard Stereotaxic Instrument, SGL M, model 68801, plus mouse adaptor 68030, supplied by 2Biol Italy), with head tilted at 90° with respect to the body. Upon incision of the skin and underlying muscles at the level of the occipital crest, cisterna magna, was exposed. A syringe equipped with a 30G needle and mounted on an automated microinjector (Quintessential Sterotaxic Injector, Product N° 53311, Stoelting, 2Biol Italy) was pre-loaded with a 250 mg/mL kaolin (Merck, K7375) solution prepared in saline. Sixty µL of kaolin solution were injected into the cisterna magna at a rate of 6 µl/min. Control animals received an equal amount of sterile saline. The duration of the experiments was 5 weeks starting from kaolin injection, following the course of the hydrocephalic pathology from the acute phase (1st week, PND28) to the chronic phase (3rd and 5th week, PND42 and PND56, respectively). The ventriculomegaly reached maximum and remained stable 3 weeks following kaolin injection.

### Evaluation of peripheral thermal hypersensitivity

Thermal hypersensitivity was assessed using the Cold plate test. With minimal animal-operator interaction, rats were removed from the cages and placed on the surface of the Cold plate (cod. 35300, Ugo Basile, Varese, Italy) maintained at a constant temperature of 4 °C ± 1 °C. Walking was limited by a cylindrical Plexiglas chamber (diameter: 10 cm, height: 15 cm). The latency time for the first appearance of a pain-related behavioural response, such as lifting or licking the paw, was recorded. The latency time limit was set at 30 s.

### Evaluation of peripheral mechanical hypersensitivity with electronic Von Frey

Mechanical hypersensitivity was measured using a dynamic plantar aesthesiometer (cod. 37550). The animals were placed individually in 20 × 20 cm plexiglass cubicles on an elevated wire floor. The tests were preceded by 30 min of acclimatization inside the cubicles. The mechanical stimulus was applied to the plantar surface of the rat’s hind paw via a von Frey filament connected to an automated mechanism placed underneath the wire floor. The paw withdrawal threshold was assessed by applying a force ranging between 0 and 5 g for tests performed one week following the injection of kaolin [18] (PND28), while between 0 and 50 g for tests performed three and five weeks following damage induction [19] (PND42 and PND56, respectively). The choice of force range was adapted to the animals’ age and physiological sensitivity at each time point. At week 1 (PND28), a range of 0–5 g was selected because both control and kaolin-injected animals produced paw withdrawal responses within this range, with kaolin animals withdrawing at significantly lower forces: a clear index of mechanical hypersensitivity. Extending the force range at this age was therefore unnecessary and would have been methodologically inappropriate. At subsequent time points (weeks 3 and 5, approximately PND42 and PND56), as animals matured, the 0–5 g range was no longer sufficient to elicit paw withdrawal responses in control animals (data not shown); the force range was therefore increased to 0–50 g to restore the dynamic window capable of capturing the difference between the physiological threshold of controls and the early withdrawal response indicative of mechanical hypersensitivity in kaolin-injected animals. Stimuli were applied to each hind paw at 5-second intervals. The threshold was defined as the minimum pressure necessary to elicit a clear and immediate paw withdrawal reflex. Voluntary movements associated with locomotion were not considered as a response.

### Evaluation of peripheral mechanical sensitivity using the Paw Pressure test

To further characterized mechanical hypersensitivity, Paw Pressure test was used. While the Von Frey test applies a light touch stimulus to the plantar surface of the hindpaw, the Paw Pressure test allows substantially higher pressure ranges to the dorsal surface of the paws, providing a complementary measure of mechanical sensitivity [20]. We used an analgesimeter (cod 37215; Ugo Basile, Varese, Italy) to perform the Paw Pressure test. Briefly, a constantly increasing pressure was applied to a small area of the dorsal surface of the hind paw using a blunt conical mechanical probe which does not hurt the animal. The instruments offer 3 force ranges, measurements in week 1 (PND28) were carried out using the 0–250 g range, while evaluations in weeks 3 (PND42) and 5 (PND56) were performed using the 0–500 g range. This choice was made to adapt the mechanical stimulus to the animal’s physiological growth. Mechanical pressure was increased until vocalization or a withdrawal reflex occurred while rats were lightly restrained. Vocalization or withdrawal reflex thresholds were expressed in grams. An arbitrary cutoff value of 100 g was adopted.

### Evaluation of cephalic thermal hypersensitivity using the orofacial evaporative cooling test

The cold pain threshold of the mandibular nerve area (V3; the mandibular nerve area) was evaluated by detecting the response to acetone-induced cold stimuli [21, 22]. Rats were given 20 min to acclimate to the testing environment before the test. Under mild restraint, the V3 skin area was treated with 50 µL of 90% acetone (diluted in distilled water) using a 100 µL pipette. Sufficient care was taken to prevent acetone from contacting the eyes, mouth, or nose. Positive reactions were defined as wiping and scratching of the V3 region by a front or hind paw. Total orofacial wiping and scratching time was measured within a cutoff of 2 min. An increase in wiping time compared to controls was defined as cold hypersensitivity. Assessment of the infraorbital area (V2: skin of the infraorbital area) was not performed, as precise delivery of acetone to this region without inadvertent ocular contact is not reliably achievable [22].

### Spontaneous pain

Spontaneous pain was evaluated using the rat grimace scale (RGS) scoring [23]. In brief, the rats were placed in individual plexiglass chambers and monitored by researchers for 30 min. Based on 4 parameters, orbital tightening, ear position, nose bulge, and whisker change, blinded experienced evaluators performed RGS scoring from 0 to 2 (0 = not present, 1 = moderate, 2 = severe). We calculated a total score as the sum of the 4 parameters for each evaluator to obtain the final RGS score.

### Open field

The open field test was conducted to assess motor alterations in rats. Briefly, rats were placed in the centre of the apparatus (100 × 100 cm) and several parameters were recorded using a video tracking system (ANY-maze, Ugo Basile, Varese, Italy) for 10 min. The open field maze was cleaned between each rat with 70% ethanol to eliminate odour [24].

### Elevated plus maze

As a measure of anxiety-like behaviour, the elevated plus maze was performed (Ugo Basile, Varese, Italy) [25]. Briefly, at the start of the test performed 1, 3 and 5 weeks after kaolin injection, rats were positioned in the central area facing one of the open arms and were allowed to explore freely for 5 min. Time spent in the centre, open arms and dark arms, and total distance travelled were measured using AnyMaze software (Ugo Basile, Varese, Italy). The test was conducted under standard overhead room lighting. The light source was positioned directly above the maze; open arms were therefore fully illuminated, while closed arms remained naturally shielded by their surrounding walls.

### Motor resistance assessment

The Rotarod apparatus (Ugo Basile, Varese, Italy) consists of a platform on which a rotating rod with a diameter of 6 cm and a non-slip surface rests. The rod was positioned at a height of 25 cm from the base and rotated at a speed of 10 rpm. The rod, 36 cm long, is divided into four equivalent sections by the interposition of five discs, so the apparatus can evaluate up to 4 rats simultaneously. The integrity of the animals’ motor resistance was assessed on the basis of the number of falls from the rod in a maximum time of 600 s. Upon reaching six falls, the test was suspended and the time recorded [26]. Fixed-speed protocols are widely employed when endurance-related outcomes are of interest. Therefore, maintaining a constant speed throughout the test allowed us to specifically evaluate the animals’ ability to sustain prolonged motor activity [19, 27]. Furthermore, the number of falls was selected as the outcome measure because animals were repeatedly returned to the rotating rod after each fall, enabling the assessment of endurance across the entire testing session rather than recording only the latency to the first fall.

### Brain water content measurement

Brain water content was determined using the wet–dry weight method. Following euthanasia, brain tissue was rapidly removed, and each sample was immediately weighed to obtain the wet weight (WW) and then placed in a drying oven at 100 °C overnight s to obtain the dry weight (DW). Brain water content is expressed as percentage of water content relative to wet weight using the following formula: [(WW – DW) / WW] × 100.

### Pharmacological testing

To characterize the type of pain associated with the model, acute pharmacological treatments were administered and their effects assessed over time. Animals received a single dose of one of the following drugs: morphine (S.A.L.A.R.S.; Como, Italy; 3 mg/kg, subcutaneous), duloxetine (Lilly, Italy; 15 mg/kg, oral gavage), paracetamol (Galeno S.r.l, Prato, Italy; 100 mg/kg, oral gavage), or acetazolamide (TEOFARMA, Pavia, Italy; 100 mg/kg, oral gavage, used as a clinical reference compound). Administration volume was set to 0.5 ml for each 100 g of animal weight. Morphine was dissolved in saline solution whereas paracetamol, duloxetine and acetazolamide were suspended in 1% carboxymethilsodium salt (CMC). Prior to drug administration, animals underwent baseline assessment of nociceptive threshold using the Paw Pressure Test and Cold Plate Test. The baseline assessment corresponds to the time 0 in the graphs. Behavioural testing was performed at 15, 30, 45, 60, and 90 min post-treatment. The Paw Pressure Test was used to evaluate mechanical hypersensitivity, while the Cold Plate Test assessed thermal hypersensitivity.

### Tissue collection and preparation

Animals were euthanized by decapitation. Heads and spines were fixed in 10% paraformaldehyde for 3 days, then brains and spinal cords were isolated, post-fixed for 24 h, dehydrated in 20% and 30% sucrose (24 h each), frozen, and stored at − 80 °C. For cryosectioning, both the brains and spinal cords were embedded in cryostat medium (for brains, Epredia™ M-1 Embedding Matrix, Fisher Scientific, ref 10056778; for spinal cord, O.C.T. Compound embedding medium for cryostat; Bioptica, ref 05-9801). Sections were cut using a cryostat (Leica, CM 1950). 20 μm coronal brain sections were obtained and mounted on polarized slides. Section selection was guided by the rat brain atlas (The Rat Brain in Stereotaxic Coordinates, 4th edition, Paxinos & Watson), focusing on the following coordinates: figure 22 (measurement of the Evans Index and immunofluorescence of lateral ventricles and ependyma); figure 28 (immunofluorescence of the choroid plexuses); figure 45 (immunofluorescence analysis in the periaqueductal gray). Spinal cord sections were cut transversely in the lumbar region at a thickness of 5 μm. Sections were collected consecutively within each target anatomical region and mounted sequentially across multiple slides, such that the spacing between sections on the same slide was sufficient to prevent overlap of cell bodies across adjacent tissue regions, minimizing the risk of pseudo replications during downstream immunofluorescence analysis. One single slide per animal was used in each immunofluorescence assay.

### Macroscopic brain damage scoring

Brain swelling and tissue integrity were evaluated at the time of skull removal using a semi-quantitative scale ranging from 0 to 3, based on the following criteria. Score 0, no visible alterations; brain tissue appeared normal at the time of skull removal. Score 1, Swollen tissue; the brain retained its overall shape upon skull removal. Score 2: Pronounced swelling; cortical collapse at the level of the ventricles was observed during skull removal. Score 3: Loss of tissue integrity; severe swelling with substantial cortical thinning and whole brain collapse upon skull removal.

### Ventricular enlargement

The Evans Index was calculated as the ratio between the maximum width of the frontal horns of the lateral ventricles and the maximum internal skull diameter measured at the level of the foramen of Monro (according to anatomical atlas reference). This parameter is used to assess ventricular enlargement and is considered indicative of ventriculomegaly when exceeding the threshold value of 0.3 [28].

### Gene expression

Quantitative Real-Time PCR (qPCR) was performed to assess the expression levels of target genes in the choroid plexus. Total RNA was extracted using (RNeasy Micro Kit, Qiagen; REF 74004) following the manufacturer’s instructions. RNA concentration and purity were determined spectrophotometrically (Accuris, SmartdropX NS1010-E). 900 ng of RNA was reverse transcribed into cDNA (Takara, RR047A). qPCR was carried out using (Biorad, CFX Connect Real-time system) and (Biorad, SsoAdvanced Universal SYBR^®^ Green Supermix, #1725274). Specific validated primers for CAIII, CAIV, GFAP, and the housekeeping gene GAPDH were purchased (Biorad, PrimePCR™ SYBR^®^ Green Assay). Reactions were performed in duplicate in a final volume of 20 µL. Gene expression was normalized on GAPDH and results are expressed as percentage changes relative to control samples.

### Immunofluorescence assays

The detailed protocol is reported in Table 1. Negative controls, incubated with secondary antibodies only, were included in all procedures to assess nonspecific antibody binding.

**Table 1:** Immunofluorescence assay protocol

Step	Materials	Temperature	Volume	Time
Washing X3	PBS 1X	Room temperature	500 µL	10 min x3
Blocking of non-specific binding sites and membrane permeabilization	Blocking solution (BSA 5% in PBS 1X + Triton 0.3%)	Room temperature	500 µL	1 h and 30 min
Primary antibodies	Aquaporin 4 Polyclonal Antibody (rabbit)Invitrogen # PA5-78812 (1:500 in blocking solution) GFAP Polyclonal Antibody (chicken) Invitrogen # PA1-10004 (1:1000 in blocking solution) Aquaporin 1 Polyclonal Antibody (chicken) Invitrogen # PA5-78806 (1:500 in blocking solution) Iba1 Antibody (rabbit) FUJIFILM Wako Pure Chemical Corporation # 016-20001 (1:250 in blocking solution)	4° C	500 µL	Overnight
Washing X3	PBS 1X	Room temperature	500 µL	10 min x3
Secondary antibodies	Goat anti-Rabbit IgG (H + L) Cross-Adsorbed Secondary Antibody, Alexa Fluor™ 488, Invitrogen # A-11,008 (1:1000 in blocking solution) Goat anti-Chicken IgY (H + L) Secondary Antibody, Alexa Fluor™ 647 Invitrogen # A-21,449 (1:1000 in blocking solution) Chicken anti-Rabbit IgG (H + L) Cross-Adsorbed Secondary Antibody, Alexa Fluor™ 647 Invitrogen # A-21,443 (1:1000 in blocking solution)	Room temperature	500 µL	3 h
Washing X4	PBS 1X	Room temperature	500 µL	10 min x4
Cover	Aqueous mounting medium + DAPI (Fluorshield; Merck F6057)	Room temperature	500 µL	4° storage up to image acquisition

### Images acquisition

Images were acquired using a Leica DM6000B fluorescence microscope equipped with a Leica DFC350FX digital camera, with a 40X objective. For each field of view for brain areas, 18 serial images were captured along the z-axis (4.97 μm Z-stack), for spinal cord, 13 steps along the z-axis (3.51 μm Z-stack). For each animal, six fields of view were collected from the somatosensory cortex and the ventricles. In the case of the periaqueductal gray, nine fields of view were acquired from the parenchyma and six from the ependymal lining of the aqueduct. Three fields of view were obtained from the choroid plexus. Four fields of view for dorsal horns of the spinal cord. Data resulting from the analysis of the technical replicates were averaged to generate a single biological replicate per animal.

### Quantification of GFAP and AQP4 fluorescence intensity

Fluorescence intensity analysis for GFAP and AQP4 was performed using ImageJ/FIJI. Raw, non-thresholded images were first opened and the individual fluorescence channels were separated. A Z-projection was then generated for each channel to obtain a representative 2D image from the z-stack. Images were converted to 8-bit. A region of interest (ROI) was manually defined for each field, carefully excluding borders, background areas, and saturated regions. An initial measurement was performed to extract parameters including mean and standard deviation of pixel gray values. A threshold was then applied, with the lower threshold value set by adding twice the standard deviation to the mean intensity measured in the ROI. A second measurement was then performed on the thresholded image, ensuring that the “Limit to threshold” option was selected. This approach minimizes the impact of background noise. Mean pixel intensity above threshold was selected as the primary outcome measure, as it provides intrinsic normalization for the immunoreactive area defined by the threshold, minimizing possible area-related confounding factors related to mechanical tissue compression. Mean pixel intensity corresponds to integrated density divided by positive area. The values of mean intensity were measured in different field of views, then averaged into a single biological replicate per animal before statistical comparison.

### Cell counting analysis

Quantification of GFAP-positive cells was performed using ImageJ with the Cell Counter plugin. Images were first split into individual fluorescence channels, keeping only the DAPI (nuclear marker) and GFAP (astrocytic marker) signals. A Z-projection was generated for both channels to obtain a representative two-dimensional image from the full z-stack. The DAPI and GFAP projections were then merged into a single image to allow simultaneous visualization of nuclei and astrocytic processes. Contrast and brightness were adjusted when necessary to enhance cellular structures and facilitate clear identification of individual cells. Manual cell counting was performed on the merged images using the Cell Counter plugin. Results were expressed as percentage of GFAP-positive cells density compared to respective control group. Volume per field of view was calculated as the product of the field of view area and the z-stack thickness (4.97 μm for brain sections and 3.51 μm for spinal cord sections). Per-animal estimates of cells/mm^3^ were obtained by averaging densities across all acquired fields of views to generate a single biological replicate, before statistical group comparison.

### Astrocytes morphology analysis

To analyze astrocyte morphology, GFAP-labelled images underwent pre-processing that included collapse of the Z-stack, merge with respective DAPI-labelled acquisitions, 8-bit transformation, contrast adjustment, noise reduction, and thresholding for segmentation. Using the Skeletonize function on ImageJ, the morphology of each astrocyte was simplified to a single-pixel-wide skeleton. The number of branches was then calculated using the AnalyzeSkeleton tool. In addition, the spatial distribution and complexity of astrocyte branching were evaluated through Sholl analysis. Concentric circles, centred on the astrocyte nucleus, were drawn at fixed intervals of 5 μm. The software calculated how many times astrocytic processes intersected each circle, generating a Sholl profile. Morphological analyses (Skeleton analysis and Sholl analysis) were performed on 12 cells per animal, sampled across multiple fields of view to ensure spatial representativeness. The value reported in the graphs for each animal represents the mean of the individual cell-level measurements, collapsed into a single biological replicate per animal. This averaging step was applied prior to any group-level statistical comparison.

### Microglial activation assessment

Quantification of Iba1-positive cells was performed using ImageJ with the Cell Counter plugin. Images were first split into individual fluorescence channels, DAPI (nuclear marker) and Iba1 (microglial marker) signals. A Z-projection was generated for both channels to obtain a representative two-dimensional image from the full z-stack. The DAPI and Iba1 projections were then merged into a single image to allow simultaneous visualization of nuclei and microglial processes. Contrast and brightness were adjusted when necessary to enhance cellular structures and facilitate clear identification of individual cells. To assess microglial activation state, Iba1-positive cells were classified as quiescent or reactive based on visual inspection of their morphological appearance on immunofluorescence images. Quiescent microglia were identified by a small cell body and a highly ramified morphology with thin, elongated processes. Reactive microglia were characterized by somatic enlargement and retraction of cellular processes. Manual cell counting was performed on the merged images using the Cell Counter plugin. For each field of view, the total number of Iba1-positive cells and the number of reactive cells were recorded. Results were expressed as Iba1-positive cell density (%) and the percentage of reactive cells over the total Iba1-positive cell count. Per-animal estimates of density (cells/mm^3^) and number of reactive cells were obtained across all acquired fields of views to generate a single biological replicate, before statistical group comparison.

### Statistical analysis

Statistical analysis was performed using OriginPro 2024b. Statistical comparisons for time course evaluation of sensory alterations, brain water content, Evan’s ratio and immunofluorescence assays were performed using the two-sided Mann–Whitney test. In acute pharmacological tests (paw pressure test and cold plate test), the same animals were evaluated repeatedly at baseline (time 0 before drugs administrations) and at time points 15, 30, 45, 60, and 90 min after drug administrations. After verification of assumption of normality (Shapiro-Wilk test on the mean residuals; observation: group mean x corresponding time), a Two-Way Repeated Measures ANOVA was performed. The between-subjects factor was Group (Control, Kaolin, Kaolin+Morphine, Kaolin+Duloxetine, Kaolin+Paracetamol, Kaolin+Acetazolamide). The within-subjects factor (repeated measures): Time (0, 15, 30, 45, 60, 90 min). Relevant significant interactions between Group * Time are reported in the graphs. Statistical comparisons for molecular data of qPCR were preceded by a Shapiro–Wilk test for normality, followed by a two-sample t-test with Welch’s correction. Correlations were assessed using Spearman’s rank correlation analysis. The first significance threshold for all tests was set for *p* < 0.05, the second threshold was set for *p* < 0.01. The numerosity for each experimental evaluation can be found in the figure legends.

## Results

### Hydrocephalus-associated sensory alterations

**Fig. 1:**
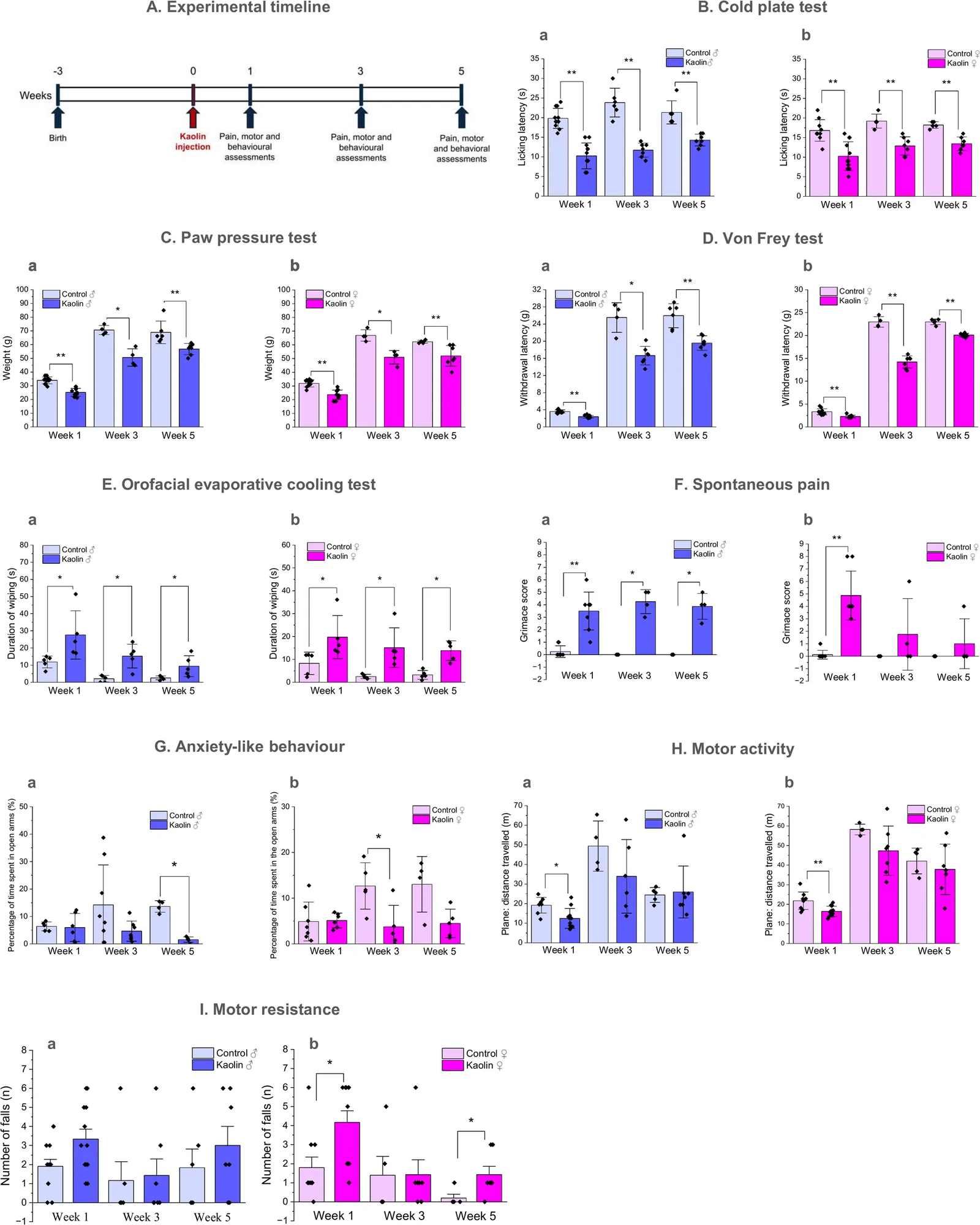
Hydrocephalus-induced pain and emotional alterations in male and female rats. **A**) Timeline of the experimental procedures. Three-weeks male and female rats were subjected to intracisternal injection of 60 µL kaolin solution (250 mg/mL) for hydrocephalus development. One, three and five weeks later, behavioural measurements were performed. The response against **B**) thermal stimulus, Cold plate test (licking latency, s) **C** - **D**) mechanical stimuli, Paw Pressure test and Von Frey test respectively (weight and withdrawal latency, respectively, both measured in g) were measured. Moreover, **E**) orofacial pain was measured with evaporative cooling test (duration of wiping, s) and **F**) spontaneous pain was assessed using the Grimace scale scoring system. **G**) Emotional alteration correlated to persistent pain like anxiety-like behaviour was evaluated by the Elevated plus maze test (percentage of time spent in the open arms, %). **H**) Motor activity and **I**) motor resistance were assessed by the Open field and Rota rod test, respectively (plane distance travelled in m and number of falls, respectively). Data are presented as mean ± SD. Statistical comparisons were performed using the two-sided Mann–Whitney test. Significance thresholds were set at *p* < 0.05 (*) and *p* < 0.01 (**). *Cold plate test*; male 1 week, *p* = 0.0000518, ctr *n* = 11, kaol *n* = 12; **Ba**; female 1 week, *p* = 0.000621, ctr *n* = 10, kaol *n* = 12, **Bb**; male 3 weeks, *p* = 0.00336, ctr *n* = 6, kaol *n* = 7, **Ba**; female 3 weeks, *p* = 0.00534, ctr *n* = 5, kaol *n* = 7, **Bb**; male 5 weeks, *p* = 0.00310, ctr *n* = 6, kaol *n* = 7, **Ba**; female 5 weeks, *p* = 0.00551, ctr *n* = 5, kaol *n* = 7, **Bb**). *Paw pressure test*; male 1 week, *p* = 0.0000690, ctr *n* = 11, kaol *n* = 12, **Ca**; female 1 week, *p* = 0.000187, ctr *n* = 10, kaol *n* = 12, **Cb**; male 3 weeks, *p* = 0.030383, ctr *n* = 4, kaol *n* = 4, **Ca**; female 3 weeks, *p* = 0.0284, ctr *n* = 4, kaol *n* = 4, **Cb**; male 5 weeks, *p* = 0.00331, ctr *n* = 6, kaol *n* = 7, **Ca**; female 5 weeks, *p* = 0.00551, ctr *n* = 5, kaol *n* = 7, **Cb**). *von Frey test*; male 1 week, *p* = 0.0000554, ctr *n* = 11, kaol *n* = 12, **Da**; female 1 week, *p* = 0.000252, ctr *n* = 10, kaol *n* = 12, **Db**; male 3 weeks, *p* = 0.0107, ctr *n* = 4, kaol *n* = 7, **Da**; female 3 weeks, *p* = 0.0107, ctr *n* = 4, kaol *n* = 7, **Db**; male 5 weeks, *p* = 0.00340, ctr *n* = 6, kaol *n* = 7, **Da**; female 5 weeks, *p* = 0.00340, ctr *n* = 5, kaol *n* = 7, **Db)**. *Orofacial evaporative cooling test;* male 1 week, *p* = 0.0122, ctr, *n* = 5; kaol, *n* = 5, **Ea**; female 1 week, *p* = 0.0122, ctr, *n* = 5; kaol, *n* = 5, **Eb**; male 3 week, *p* = 0.0119, ctr, *n* = 5; kaol, *n* = 5, **Ea**; female 3 week, *p* = 0.0122, ctr, *n* = 5; kaol, *n* = 5, **Eb**; male 5 week, *p* = 0.0278, ctr, *n* = 5; kaol, *n* = 5, **Ea**; female 5 week, *p* = 0.0122, ctr, *n* = 5; kaol, *n* = 5, **Eb**. *Grimace score*; male 1 week, *p* = 0.000883, ctr *n* = 8, kaol *n* = 8, **Fa**; female 1 week, *p* = 0.000443, ctr *n* = 8, kaol *n* = 8, **Fb**; male 3 weeks, ctr *n* = 4, kaol *n* = 4, *p* = 0.0202, **Ea**; female 3 weeks, ctr *n* = 4, kaol *n* = 4, **Fb**; male 5 weeks, *p* = 0.0202, ctr *n* = 4, kaol *n* = 4, **Fa**; female 5 weeks, ctr *n* = 4, kaol *n* = 4, **Fb**. *Elevated plus maze*,* open arms time*; male 1 week, ctr *n* = 6, kaol *n* = 7, **Ga**; female 1 week, ctr *n* = 7, kaol *n* = 8, **Gb**; male 3 weeks, ctr *n* = 9, kaol *n* = 7, **Ga**; female 3 weeks, *p* = 0.0357, ctr *n* = 5, kaol *n* = 6, **Gb**; male 5 weeks, *p* = 0.0139, ctr *n* = 6, kaol *n* = 6, **Ga**; female 5 weeks, ctr *n* = 4, kaol *n* = 5, **Gb**. *Open field test*; male 1 week, *p* = 0.0117, ctr *n* = 8, kaol *n* = 11, **Ha**; female 1 week, *p* = 0.00493, ctr *n* = 9, kaol *n* = 11, **Hb**; male 3 weeks, ctr *n* = 4, kaol *n* = 6, **Ha**; female 3 weeks, ctr *n* = 4, kaol *n* = 7, **Hb**; male 5 weeks, ctr *n* = 5, kaol *n* = 7, **Ha**; female 5 weeks, ctr *n* = 5, kaol *n* = 7, **Hb**. *Rotarod test*; male 1 week, ctr *n* = 11, kaol *n* = 12, **Ia**; female 1 week, *p* = 0.0103, ctr *n* = 10, kaol *n* = 12, **Ib**; male 3 weeks, ctr *n* = 6, kaol *n* = 7, **Ia**; female 3 weeks, ctr *n* = 5, kaol *n* = 7, **Ib**; male 5 weeks, ctr *n* = 6, kaol *n* = 7, **Ia**; female 5 weeks, *p* = 0.0352, ctr *n* = 5, kaol *n* = 7, **Ib**

Three weeks old male and female rats underwent injection of kaolin solution (60 µL; 250 mg/mL) in the cisterna magna to induce hydrocephalus. Pain and motor alterations as well as anxiety and depressive-like behaviours were assessed 1, 3 and 5 weeks after kaolin injection (Fig. 1A) (corresponding to PND28, PND42 and PND56, respectively). Statistical analyses of behavioural data were performed using a non-parametric approach (two-sided Mann–Whitney test), comparing kaolin-injected animals with their sex- and timepoint-matched control group at each individual timepoint. Peripheral and cephalic somatic pain associated with hydrocephalus was evaluated using thermal and mechanical stimuli through the Cold plate, Paw pressure, von Frey and orofacial evaporative cooling tests. With no sex differences, animals developed peripheral and cephalic thermal hypersensitivity (Fig. 1B and E) and peripheral mechanical hypersensitivity (Fig. 1C and D) at all time points evaluated suggesting a correlation between hydrocephalus and sensory alterations. Cold stimuli applied on paws, led to an increased time in licking latency in hydrocephalic rats, a pain-related behaviour index of peripheral thermal hypersensitivity. Moreover, animals with kaolin-induced hydrocephalus displayed a prolonged aversive response following acetone application, compared to control animals, characterized by more frequent and longer wiping and scratching bouts. Of note, at week 1 (PND28), likely due to the young age of the animals, control rats also exhibited a relatively prolonged aversive response duration; this normalized at weeks 3 (PND42) and 5 (PND56), where control scores stabilized. Hydrocephalic animals consistently showed increased aversive behavior at all time points evaluated, confirming the presence of orofacial thermal hypersensitivity. The mechanical thresholds observed in control animals at week 1 post-injection (PND28) are consistent with the well-established developmental maturation of nociceptive thresholds in rats, which increase progressively with postnatal age [29]. Our control animals at week 1 display thresholds in the lower range of this developmental trajectory, as expected. Nonetheless, hydrocephalic rats consistently displayed a lowered mechanical threshold compared to the respective sex- and age-matched controls, confirming the presence of peripheral mechanical hypersensitivity. Moreover, both male and female rats showed the onset of spontaneous pain as early as one week after the surgical procedure for kaolin injection, compared to untreated animals (control group). Although this condition of spontaneous pain persisted in male rats at later time points (weeks 3 and 5; Fig. 1Fa), it gradually decreased in female rats, no longer reaching statistical significance (Fig. 1Fb).

We also assessed the emotional components frequently associated with neuropathic pain conditions [30]. In the Elevated plus maze test, no significant alterations were observed in hydrocephalic male rats regarding the distance travelled within the arena during the 5-minute session (Supplementary Figure S2A). In contrast, kaolin-injected female rats showed a statistically significant reduction in this parameter at week 3 compared to controls (Supplementary Figure S2B). At this time point, female rats displayed a pronounced anxiety-like phenotype, as evidenced by a significant reduction in the number of entries, time spent, and mobility in the centre and open arms of the maze, accompanied by an increase in time spent in the closed arms (Fig. 1Gb, Representative trajectory plots Supplementary Figure S3; Supplementary Figure S4M, N and O). Male kaolin-injected rats didn’t exhibited a robust anxiety-like behaviour at week 3 (Fig. 1Ga), with later onset on week 5, with a reduction in entries, mobility time, and time spent in the open arms compared to control animals (Fig. 1Ga, Representative trajectory plots Supplementary Figure S3; Supplementary Figure S4H) and increased time spent in the dark arms (Supplementary Fig. 4I). The final set of behavioural measurements aimed at investigating potential motor alterations and motor dysfunctions associated with hydrocephalus. In the Open Field test, both male and female kaolin-injected animals exhibited a significant reduction in the total distance travelled within the arena compared to controls, as early as one-week post-surgery. However, this alteration was no longer evident at later time points (weeks 3 and 5; Fig. 1G; Representative trajectory plots Supplementary Figure S5). Comparable results were obtained in the Rotarod test, which showed an increased number of falls from the rotating rod in both sexes of kaolin-injected rats at one-week post-injection (Fig. 1H). Notably, this motor deficit persisted only in female hydrocephalic rats at week 5 (Fig. 1Hb).

### Dysregulation of brain fluids homeostasis correlates with alteration in pain threshold

**Fig. 2:**
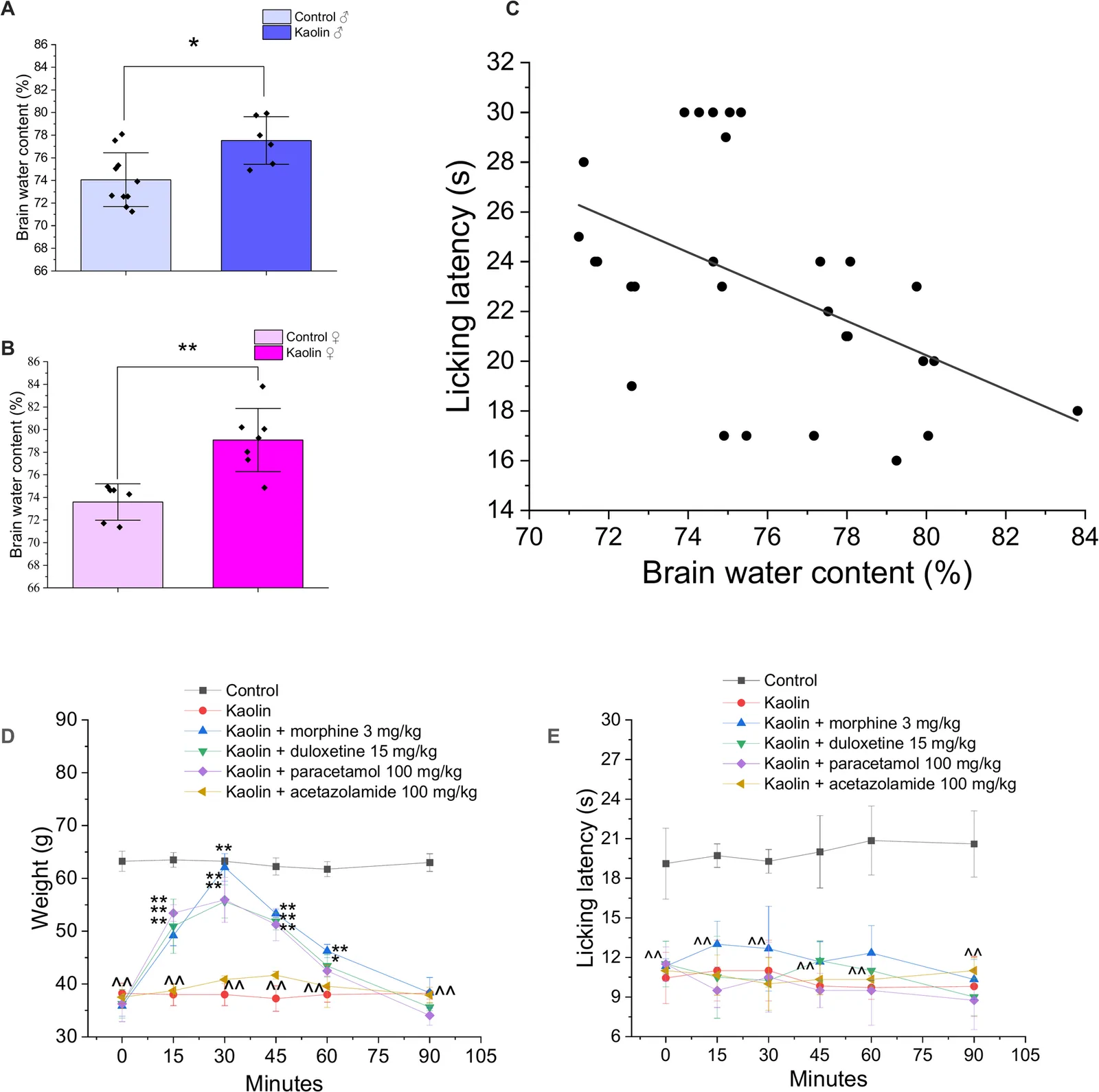
Dysregulation of brain fluids homeostasis correlates with alteration in pain threshold. Brain water content percentage in control and kaolin-induced hydrocephalus animals, male (**A**, *p* = 0.0261, ctr *n* = 10, kaol *n* = 6) and female (**B**, *p* = 0.00534, ctr *n* = 6, kaol *n* = 7). Data are presented as mean ± SD. Statistical comparisons were performed using the two-sided Mann–Whitney test. Significance thresholds were set at *p* < 0.05 (*) and *p* < 0.01 (**). **C**) The correlation between brain water content and pain threshold was assessed using Spearman’s rank correlation. A significant negative correlation was observed (ρ = − 0.52198, *p* = 0.00368). **D**) Impact on mechanical sensitivity and **E**) thermal sensitivity of reference compounds were evaluated measuring the animals’ response mechanical stimulus (Paw Pressure test, weight measured in g) and thermal stimulus (Cold plate test, licking latency in s), respectively. Duloxetine (15 mg/kg), paracetamol (100 mg/kg) and acetazolamide (100 mg/kg) were suspended in 1% carboxymethylcellulose (CMC) and administered *per os*. Morphine (3 mg/kg) was dissolved in sterile saline solution and subcutaneously injected. Behavioural assessments were performed at baseline (time 0 before the drugs administrations) and 15, 30, 45, and 60 min after treatments. Data are presented as mean ± SD. Statistical analysis was performed using Two-way ANOVA for Repeated Measure followed by Bonferroni corrections for multiple comparisons, preceded by Shapiro-Wilk test for normality on mean residuals. Factor A = Group, Factor B = Time. Relevant significant interactions between Group*Time are shown in the graphs with significance thresholds set at *p* < 0.05 (*) and *p* < 0.01 (**) vs. kaolin. *p* < 0.01 (^^) vs. control. Numerosity; Control group *n* = 5 (2 males and 3 females). Kaolin group *n* = 5 (3 males and 2 females). Kaolin + morphine 3 mg/kg group *n* = 3 (2 males and 1 female). Kaolin + duloxetine 15 mg/kg *n* = 4 (1 male, 3 females). Kaolin + paracetamol 100 mg/kg *n* = 4 (2 males and 2 females). Kaolin + acetazolamide 100 mg/kg *n* = 3 (2 males and 1 female)

Brain water content was determined in control and hydrocephalic rats using the wet-dry method, as an index of parenchymal fluid uptake and accumulation, cardinal sign of hydrocephalus pathology. Animals with hydrocephalus displayed significantly higher parenchymal fluid content in comparison to control animals (Fig. 2A and B). Brain water content statistical comparison was performed using two-sided Mann-Whitney test. Increased brain water content significantly correlated with reduction in pain threshold (Fig. 2C). The correlation analysis was specifically designed to be performed on animals sacrificed for brain water content measurement, which required a dedicated cohort. A single behavioural readout (thermal hypersensitivity assessed through Cold Plate test) was selected as a representative measure of the overall pain phenotype. Behavioural data recorded in this animal cohort are available in the supplementary material (Supplementary Fig. 6). Correlation was assessed using Spearman’s rank correlation analysis. To investigate the type of pain characterizing the model, acute pharmacological tests were performed using morphine (3 mg/kg, subcutaneous), duloxetine (15 mg/kg, oral gavage), and paracetamol (100 mg/kg, oral gavage), and compared with the clinical reference compound acetazolamide (100 mg/kg, oral gavage). Pharmacological testing was performed on a mixed-sex cohort: the control group comprised 5 animals (2 males and 3 females), the kaolin group 5 animals (3 males and 2 females), the kaolin + morphine group 3 animals (2 males and 1 female), the kaolin + duloxetine group 4 animals (1 male and 3 females), the kaolin + paracetamol group 4 animals (2 males and 2 females), and the kaolin + acetazolamide group 3 animals (2 males and 1 female). The pharmacological results were analysed using a Two-Way ANOVA for Repeated Measure design (following verification of assumption of normality on the mean residuals using Shapiro-Wilk test). The two factors were Group (between subjects) and Time (within subjects). Relevant significant interactions between factors are reported in the graphs. A baseline pre-test before administering the drugs was conducted to assess initial nociceptive thresholds using mechanical and thermal stimuli. Behavioural testing was then carried out at 15, 30, 45, 60, and 90 min post-administration. Among the treatments, morphine produced the most pronounced effect on mechanical sensitivity, as measured by the Paw Pressure test (Fig. 2D), with a peak effect observed at 30 min. Duloxetine and paracetamol also induced a significant increase in pain threshold compared to the kaolin group, although their efficacy was lower than that of morphine. Acetazolamide was ineffective. None of the tested drugs demonstrated any effect on thermal sensitivity at any time point, as assessed by the Cold Plate test (Fig. 2E).

### Molecular and histological alterations of brain ventricles, ependyma and choroid plexus

**Fig. 3:**
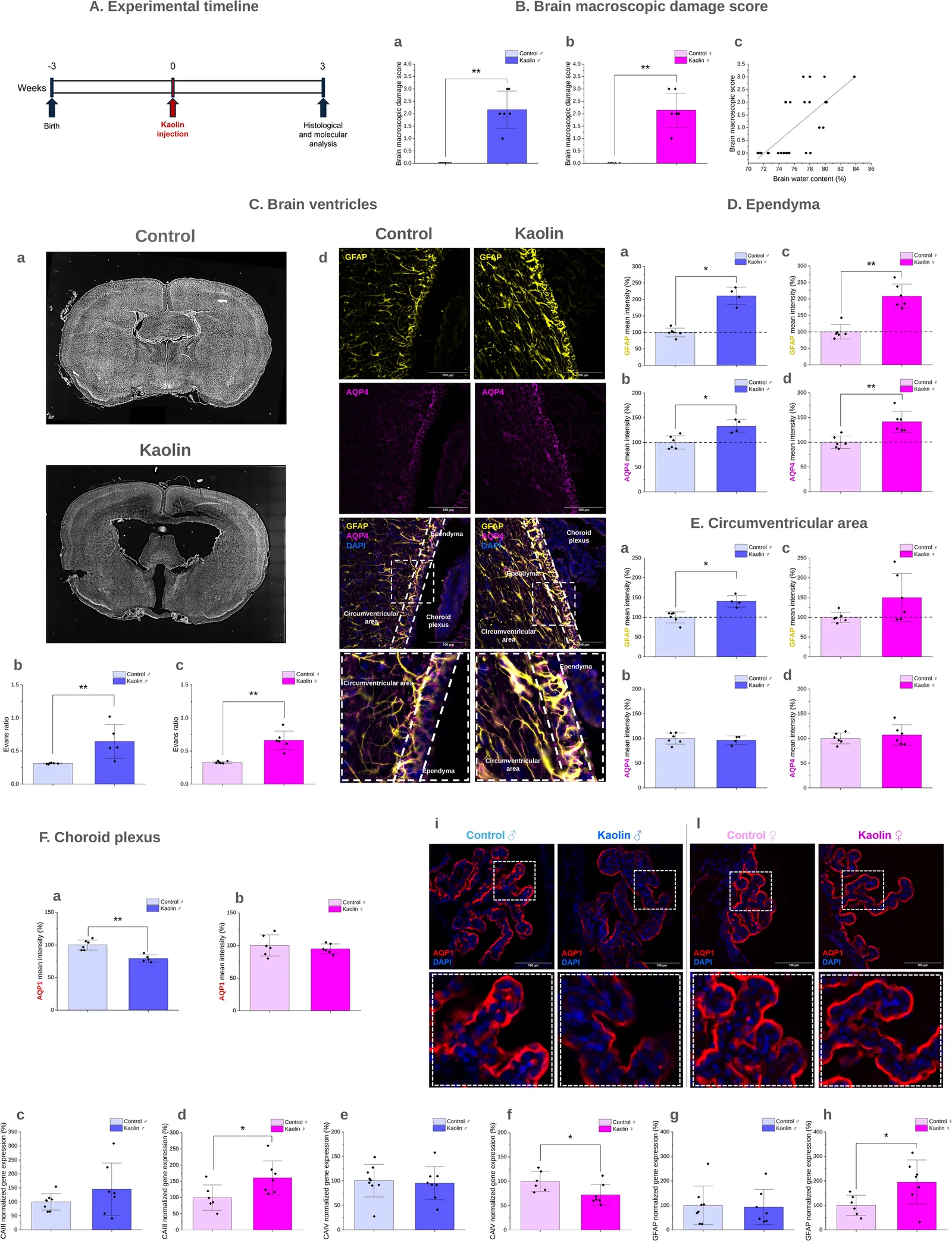
Impaired fluid homeostasis: molecular and histological alterations of brain ventricles, ependyma and choroid plexus. **A**) Experimental timeline: histological and molecular analyses were performed three weeks following kaolin injection. **B**) Brain macroscopic damage score in control and kaolin-treated animals, males (ctr *n* = 10, kaol *n* = 6, *p* = 0.000220) (**Ba**) and females (ctr *n* = 6, kaol *n* = 7, *p* = 0.00172) (**Bb**). Data are presented as mean ± SD. Statistical comparisons were performed using the two-sided Mann–Whitney test, with significance thresholds set at *p* < 0.05 (*) and *p* < 0.01 (**). **Bc**) The correlation between brain macroscopic damage score and brain water content (measured on the same animals) was assessed using Spearman’s rank correlation. A significant positive correlation was observed (ρ = 0.69147, *p* < 0.0001). The analysis of brain ventricles includes: **C**) Evans’ ratio calculated on ex vivo sections (**Ca**, representative image from male rat), in control and kaolin-treated animals, males (ctr *n* = 6, kaol *n* = 5, *p* = 0.00811) (**Cb**) and females (ctr *n* = 6, kaol *n* = 6, *p* = 0.00507 (Cc), and immunofluorescence analysis (**Cd**) in ependymal layer (**D**) for GFAP and AQP4 in control and hydrocephalic animals males (ctr *n* = 6, kaol *n* = 4, GFAP *p* = 0.0142, **Da**; AQP4 *p* = 0.0142, **Db**), and females (ctr *n* = 6, kaol *n* = 6, GFAP, *p* = 0.00507, **Dc**; AQP4, *p* = 0.00507, **Dd**), and in (**E**) circumventricular area for males (ctr *n* = 6, kaol *n* = 4, GFAP, *p* = 0.0142, **Ea**; AQP4, **Eb**) and females (ctr *n* = 6, kaol *n* = 6, GFAP **Ec**; AQP4, **Ed**). The analysis on choroid plexus (**F**) includes: immunofluorescence for AQP1 in control animals and animals with hydrocephalus, males (ctr *n* = 6, kaol *n* = 5, *p* = 0.00811, **Fa**, **Fi**,** Fl**) and females (ctr *n* = 6, kaol *n* = 6, **Fb**), and qPCR on CAIII, CAIV and GFAP transcripts in male (ctr *n* = 8, kaol *n* = 7, **Fc** and **Fg**; ctr *n* = 9, kaol *n* = 8, **Fe**) and females (ctr *n* = 6, kaol *n* = 7, CAIII, *p* = 0.0361 **Fd**; CAIV, *p* = 0.03836, **Ff**, GFAP, *p* = 0.0354, **Fh**). Choroid plexus immunofluorescence and qPCR data are presented as mean ± SD. Statistical comparisons in immunofluorescence data were performed using the two-sided Mann–Whitney test, with a significance threshold set at *p* < 0.05 (*). Statistical analysis of qPCR data was preceded by a Shapiro–Wilk test for normality, followed by a two-sample t-test with Welch’s correction. Significance thresholds were set at *p* < 0.05 (*) and *p* < 0.01 (**). *Notes* The images shown in the figures panels to illustrate immunofluorescence data were processed using the Thunder algorithm (Leica Software) to reduce background noise. Entire Thunder-processed images were subsequently adjusted for contrast and brightness to improve presentation clarity. Original unprocessed files and raw data are available upon request

Histological assessment was performed in the third week following kaolin injection (Fig. 3A). The 3-week timepoint was selected because, in our experimental setting as well as in the reference model we followed [11], ventriculomegaly reaches its maximum extent and remains stable at this stage. Moreover, motor function is preserved in our experimental setting, a good prerequisite for ensuring that pain and emotional findings are not confounded by motor deficits, therefore providing a good correlation between histological findings and behavioural outcomes. Macroscopic evaluation of brain tissue revealed a marked deterioration in appearance and structural integrity, with significantly higher macroscopic damage scores observed in kaolin-injected animals compared to controls (Fig. 3Ba and Bb). The macroscopic score showed a significant positive correlation with brain water content (Fig. 3Bc), providing evidence of the validity of the scoring system here developed. Correlation was assessed using Spearman’s rank correlation analysis.

On DAPI-stained brain sections, Evans ratio was calculated to determine ventricular enlargement. Kaolin-treated animals showed marked ventricular enlargement, cortical thinning, and parenchymal compression at the level of foramen of Monro, in line with existing literature [31], then exhibiting higher Evans Index values compared to controls, with control animals showing values around 0.3 and kaolin-treated animals displaying values equal to or greater than 0.5 (Fig. 3Ca, Cb and Cc).

In immunofluorescence analysis, we chose mean pixel intensity above threshold as the primary quantitative parameter for immunofluorescence analysis, to avoid area compression bias. In our approach, a threshold was applied to each image to define the immunoreactive area, isolating pixels with specific fluorescent signal from background. Within this threshold-defined region of interest, mean intensity reflects the average signal strength, thereby providing an intrinsic normalization for the size of the positive region. In our experimental model, the progressive ventricular enlargement and mechanical compression of the parenchyma may affect the absolute size of the immunoreactive area independently of changes in protein expression. Integrated density (defined as the product of mean intensity and positive area) incorporates both variables simultaneously and is therefore more susceptible to area-related confounding factors in this context. Mean pixel intensities are shown in the main figures. All additional quantitative parameters, including integrated density, area positive, and area FOV, are now reported in the Supplementary Materials (Supplementary Figures S7-S11), consistently show the same directional findings as mean intensity, supporting the robustness of our conclusions. Immunofluorescence analysis on lateral ventricles was performed using GFAP labelling -which stains both ependymal cells and astrocytes- and AQP4, as marker of astrocytic and ependymal functionality. In the ependymal layer lining the lateral ventricles (Fig. 3Cd), a significant increase in the expression of both GFAP and AQP4 was observed (Fig. 3Da-d). In the surrounding periventricular parenchyma, GFAP expression was upregulated -significantly in males and showing a trend toward increase in females- whereas AQP4 levels did not show a significant change (Fig. 3Ea-d). The choroid plexus did not display any distinct GFAP or AQP4 immunoreactivity.

The choroid plexus was analysed by immunofluorescence for the expression of AQP1 (Fig. 3Fa, Fb, Fi, Fl), a functional marker associated with CSF production. The analysis revealed a significant decrease in fluorescence intensity in males, while no change was observed in females. Further analysis of other molecular effectors through real-time PCR assay of CAIII, CAIV, and GFAP transcripts showed significant modulation in females, specifically upregulation of CAIII and GFAP and downregulation of CAIV, while no alterations were observed in males (Fig. 3Fc-h).

All histological and molecular data from brain ventricles and choroid plexus were analyzed using two-sided Mann-Whitney test, except molecular data of qPCR, that were analyzed with a parametric t-test following normality assumption verification.

### Altered astrocytic profile in CNS pain-controlling areas

**Fig. 4:**
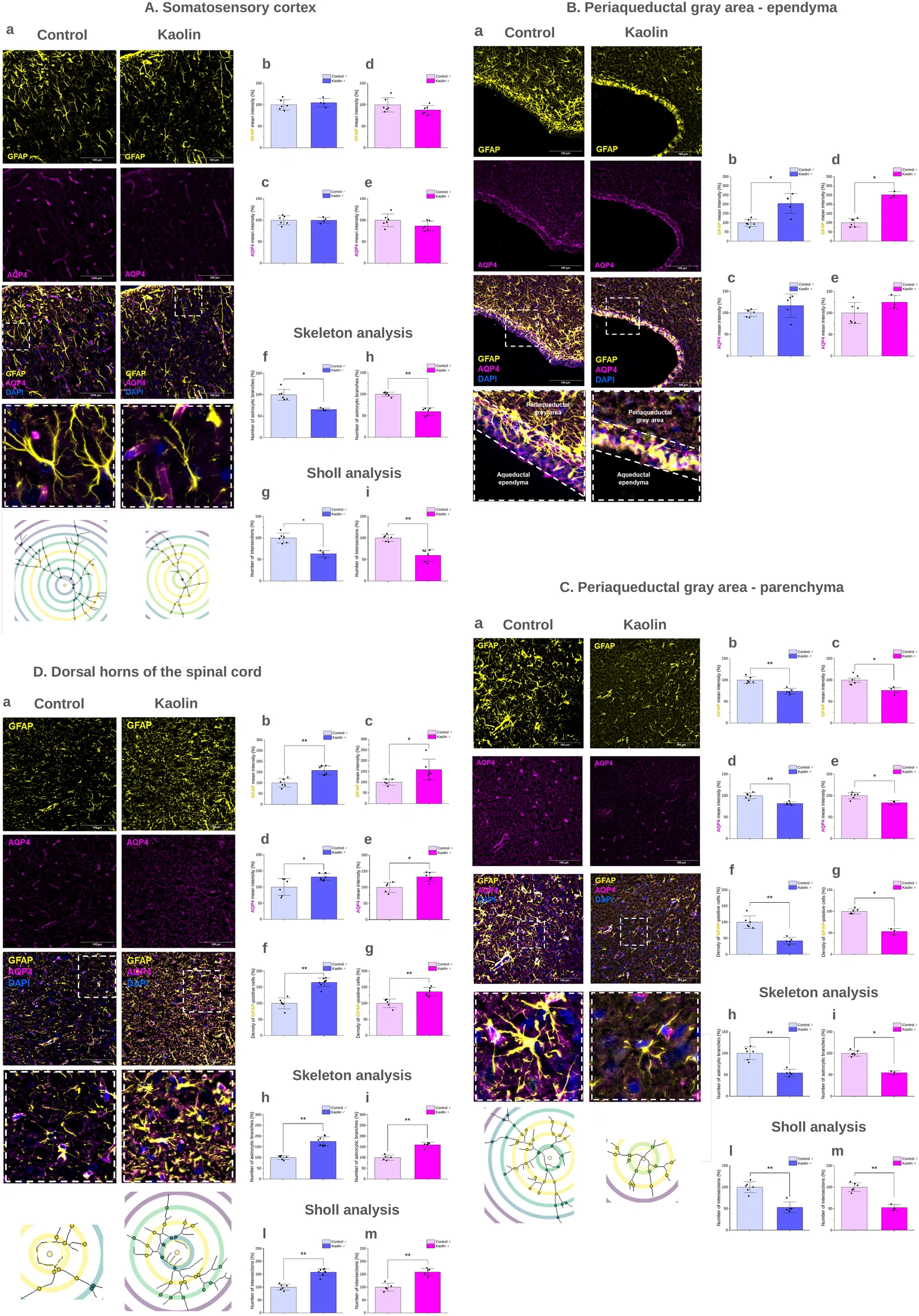
Pain-controlling CNS structures are challenged by impaired CSF circulation: area-dependently altered astrocytic profile. Immunofluorescence analysis on somatosensory cortex (**Aa**) in control and kaolin-treated rats includes: fluorescence intensity of GFAP and AQP4 in males (GFAP, **Ab**; AQP4, **Ac**) and females (GFAP, **Ad**; AQP4, **Ae**) control and kaolin-treated animals, and morphological analysis with skeleton analysis and sholl analysis in male (skeleton, *p* = 0.0142, **Af**; sholl, *p* = 0.0142, **Ag**) and female (skeleton, *p* = 0.00499, **Ah**; sholl, *p* = 0.00507, **Ai**). Numerosity for somatosensory cortex: male ctr *n* = 6, male kaol *n* = 4, female ctr *n* = 6, female kaol *n* = 6. Immunofluorescence analysis on periaqueductal gray examined aqueductal ependyma (**Ba**) and periaqueductal parenchyma (**Ca**) in control and kaolin-treated rats including: fluorescence intensity of GFAP and AQP4 in aqueductal ependymal layer of male (GFAP, *p* = 0.0137, **Bb**; AQP4, **Bc**) and female (GFAP, *p* = 0.0281, **Bd**; AQP4, **Be**), fluorescence intensity of GFAP and AQP4 in periaqueductal parenchyma of male (GFAP, *p* = 0.00507, **Cb**; AQP4, *p* = 0.00811, **Cd**) and female (GFAP, *p* = 0.0281, **Cc**; AQP4, *p* = 0.0281, **Ce**), density of GFAP-positive cells in male (*p* = 0.00811, **Cf**) and female (*p* = 0.0282, **Cg**), morphological analysis with skeleton analysis and sholl analysis in male (skeleton, *p* = 0.00811, **Ch**; sholl, *p* = 0.00811, **Cl**) and female (skeleton, *p* = 0.0281, **Ci**; sholl, *p* = 0.0281, **Cm**). Numerosity of periaqueductal gray area: male ctr *n* = 6, male kaol *n* = 5, female ctr *n* = 6, female kaol *n* = 3. Immunofluorescence analysis of dorsal horns of the spinal cord (**Da**) in control and kaolin-treated rats includes: fluorescence intensity of GFAP and AQP4 in male (GFAP, *p* = 0.00241 **Db**; AQP4, *p* = 0.0238, **Dd**) and female (GFAP, *p* = 0.0224, **Dc**; AQP4, *p* = 0.0224, **De**) rats, density of GFAP-positive cells in male (*p* = 0.00239, **Df**) and female (*p* = 0.00811, **Dg**). Morphological analysis with skeleton analysis and sholl analysis in male (skeleton, *p* = 0.00241, Dh; sholl, *p* = 0.00241, Dl) and female (skeleton, *p* = 0.00811, Di; sholl, *p* = 0.00811, Dm). Numerosity for dorsal horns of the spinal cord: male ctr *n* = 6, male kaol *n* = 8, female ctr *n* = 5, kaol *n* = 6. Data are presented as mean ± SD. Statistical comparisons were performed using the two-sided Mann–Whitney test. Significance thresholds were set at *p* < 0.05 (*) and *p* < 0.01 (**). *Notes* The images shown in the figures panels to illustrate immunofluorescence data were processed using the Thunder algorithm (Leica Software) to reduce background noise. Entire Thunder-processed images were subsequently adjusted for contrast and brightness to improve presentation clarity. Original unprocessed files and raw data are available upon request

Immunofluorescence analysis in the somatosensory cortex (Fig. 4Aa) for the markers GFAP and AQP4 revealed no significant differences between control and hydrocephalic conditions in the quantitative assessment of fluorescence intensity levels for either GFAP or AQP4 (Fig. 4Ab-e). However, qualitative analysis of astrocyte morphology showed noteworthy alterations: skeleton analysis revealed a significant reduction in the number of branches in kaolin-treated animals (Fig. 4Af, Ah), and Sholl analysis indicated a significant decrease in the number of intersections (Fig. 4Ag, Ai).

In the periaqueductal gray (Fig. 4Ba), the ependymal cell layer and the surrounding parenchymal area (Fig. 4Ca) were analysed separately. At the ependymal level, a significant increase in GFAP fluorescence intensity was observed in hydrocephalic brain sections (Fig. 4Bb, Bd), along with a trend toward increased AQP4 expression (Fig. 4Bc, Be). In the parenchymal region, a significant reduction in fluorescence intensity for both GFAP (Fig. 4Cb, Cc) and AQP4 (Fig. 4Cd, Ce) in animals with hydrocephalus was detected. Additionally, in kaolin-treated animals a decrease in the density of GFAP-positive cells (Fig. 4Cf and Cg; cell density represents the ratio of positive cell count and volume; cells/mm^3^; % change compared to respective controls are displayed in the graphs), a reduction in astrocytic branching as determined by skeleton analysis (Fig. 4Ch, Ci), and a reduction in the number of intersections from Sholl analysis (Fig. 4Cl, Cm) were observed. Overall, these data point toward a hypothesis of primary compression damage induced by ventricular enlargement, leading to cellular distress.

In the dorsal horns of the spinal cord (Fig. 4Da), fluorescence intensity of both GFAP (Fig. 4Db, Dc) and AQP4 (Fig. 4Dd, De) was increased in hydrocephalic animals, along with an increase in the density of GFAP-positive cells (Fig. 4Df, Dg). Skeleton analysis showed an increase in the number of branches (Fig. 4Dh, Di), and Sholl analysis demonstrated a higher number of intersections in kaolin-treated animals compared to controls (Fig. 4Dl, Dm). Collectively, these findings reflect increased structural complexity of spinal cord astrocytes, a hallmark of cellular activation and a finding fully consistent with a central sensitization profile.

All astrocytes data were analyzed using two-sided Mann-Whitney test.

### Microglial activation in CNS pain-controlling areas

**Fig. 5:**
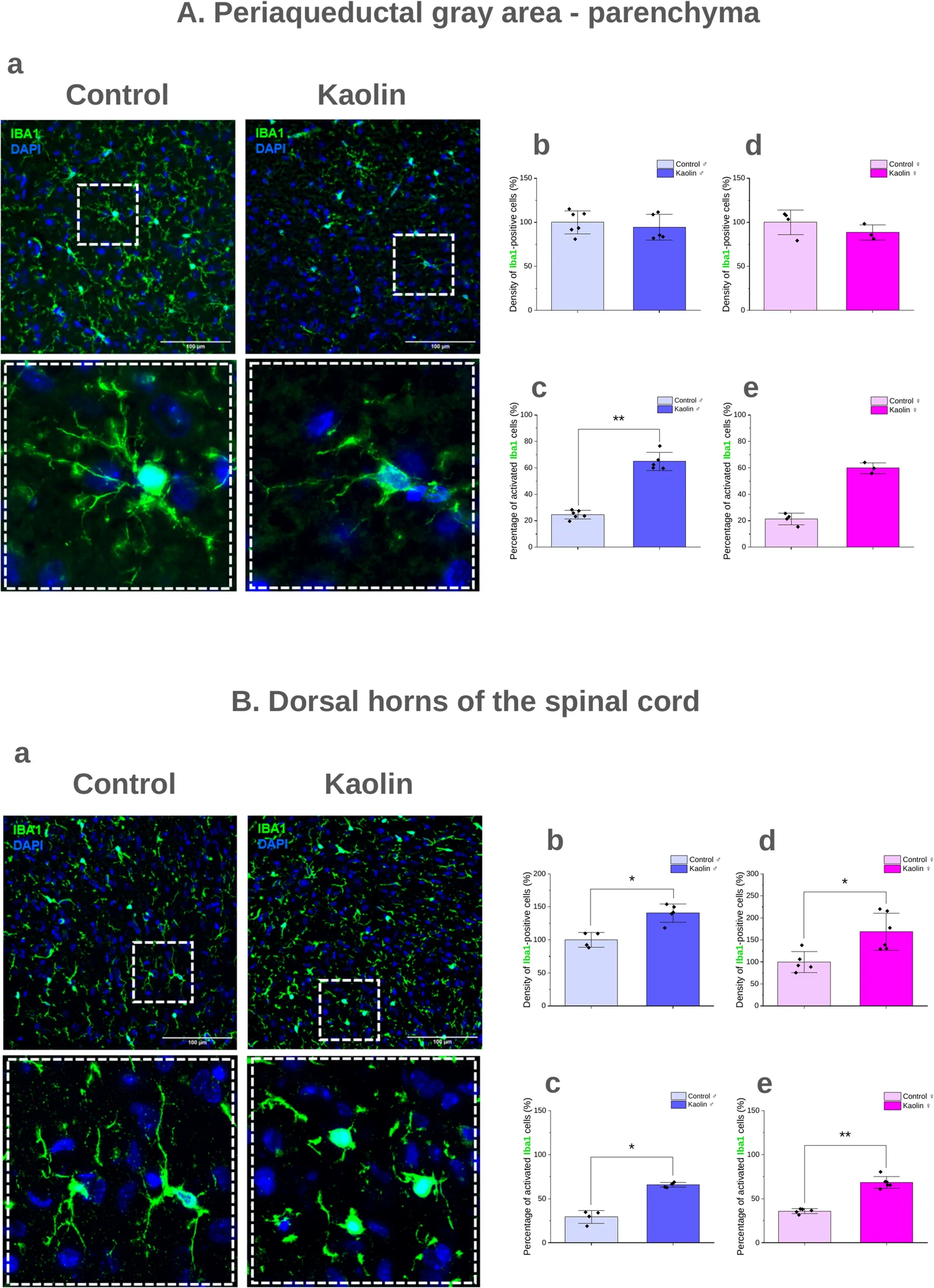
Increased microglial activation in PAG and spinal cord. **A**) Immunofluorescence analysis on periaqueductal gray area – parenchyma (**Aa**). Density of Iba1-positive cells (%) in male (*p* = 0.583; **Ab**) and female (*p* = 0.377 **Ad**) rats. Percentage of activated Iba1-positive cells (%) in male (*p* = 0.00811; **Ac**) and female (*p* = 0.05183; **Ae**) rats. Numerosity: Control male *n* = 6; Kaolin male *n* = 5; Control female *n* = 4; Kaolin female *n* = 3). **B)** Immunofluorescence analysis on dorsal horns of the spinal cord (**Ba**). Density of Iba1-positive cells (%) in male (*p* = 0.0200; **Bb**) and female (*p* = 0.0225; **Bd**) rats. Percentage of activated Iba1-positive cells (%) in male (*p* = 0.0200; Bc) and female (*p* = 0.00811; **Be**) rats. Numerosity: Control male *n* = 4; Kaolin male *n* = 5; Control female *n* = 5; Kaolin female *n* = 6). Data are presented as mean ± SD. Statistical comparisons were performed using the two-sided Mann–Whitney test. Significance thresholds were set at *p* < 0.05 (*) and *p* < 0.01 (**). *Notes* The images shown in the figures panels to illustrate immunofluorescence data were processed using the Thunder algorithm (Leica Software) to reduce background noise. Entire Thunder-processed images were subsequently adjusted for contrast and brightness to improve presentation clarity. Original unprocessed files and raw data are available upon request

To further characterize the pathological profile of kaolin-induced hydrocephalus, the contribution of microglia as a mediator of central sensitization [8] and neuroinflammation [9] was explored by Iba1 immunofluorescence in the periaqueductal gray parenchyma and the dorsal horns of the spinal cord (Fig. 5). Microglial cells were classified as resting or activated based on qualitative evaluation of their immunofluorescence appearance: resting microglia displayed a ramified morphology with long, highly branched processes, while activated microglia were identified by cell body hypertrophy and marked reduction in process length and complexity. In the PAG, the density of Iba1-positive cells did not differ significantly between control and kaolin-treated animals in either males (*p* = 0.583; Fig. 5Ab) or females (*p* = 0.377; Fig. 5Ad). As for astrocytes analysis, cell density represents the ratio of positive cell count and volume; cells/mm^3^. Data in the graphs represent the percentual change in cell density compared to respective control groups. The percentage of morphologically activated Iba1-positive cells was significantly increased in kaolin-treated males (*p* = 0.00811; Fig. 5Ac), with a trend toward increase in females (*p* = 0.05183; Fig. 5Ae), suggesting a shift toward an activated microglial phenotype in the absence of overt cell proliferation. In the dorsal horns of the spinal cord, kaolin-treated animals showed a significant increase in the density of Iba1-positive cells in both males (*p* = 0.0200; Fig. 5Bb) and females (*p* = 0.0225; Fig. 5Bd), accompanied by a significant increase in the percentage of activated microglia in both sexes (male: *p* = 0.0200, Fig. 5Bc; female: *p* = 0.00811, Fig. 5Be), indicating microglial proliferation and activation consistent with a central sensitization profile.

All microglia data were analyzed using two-sided Mann-Whitney test.

## Discussion

Hydrocephalus-associated pain is a pathological aspect that is often neglected in the clinical practice and not sufficiently addressed in animal studies, despite representing an impactful condition in the patient’s clinical presentation [4]. This issue is not exclusive to hydrocephalus, as chronic pain is also associated with other pathologies characterized by CSF disturbance, such as idiopathic intracranial hypertension [10]. Therefore, the primary aim of this work was to characterize persistent pain in an experimental model of CSF dynamics dysfunction, providing coordinates for the preclinical exploration of behavioural, pharmacological, molecular and cellular components, while simultaneously deepening the role of glial cells in pathogenetic mechanisms. Data from male and female rats were analysed separately, relative to their own control groups, as an exploratory approach to examine potential sex-related patterns in hydrocephalus-associated pain, a dimension that has been largely neglected in the preclinical literature.

Using a widely studied and validated preclinical model, specifically hydrocephalus induced by intracisternal injection of kaolin [32], we characterized the animals’ somatosensory alterations by conducting a time-course study. The results clearly demonstrate that kaolin-induced hydrocephalus leads to a significant alteration in the response threshold to thermal and mechanical stimuli applied on head and paws. These observations suggest the presence of central and peripheral sensory disturbances, with persistent peripheral and orofacial hypersensitivity associated with hydrocephalus, together with spontaneous pain (Fig. 1). These findings support the translational relevance of the model with respect to pain phenotype, incorporating both the headache component and the peripheral sensory alterations that characterized hydrocephalus-associated pain. We outlined a significant correlation between an increase in cerebral fluid content -a distinctive sign of hydrocephalic pathology- and reduction in pain threshold (Fig. 2C). Pharmacological investigations (Fig. 2D and E) have shown that pain is mixed in nature, both nociceptive, responding to morphine and paracetamol, and neuropathic, responding to duloxetine. Acute treatments have shown limited efficacy, with partial efficacy in the mechanical sensitivity, but not thermal, but with superior efficacy in comparison to the clinical reference acetazolamide. It should be noted that the dose of morphine used in this study was lower than the conventionally employed doses for analgesia induction [33, 34] since signs of toxicity, including catatonia, were observed at standard doses. The pharmacological testing presented in this study was designed as an acute characterization experiment, aimed at probing the nature of the pain phenotype (nociceptive vs. neuropathic) rather than evaluating a therapeutic regimen for hydrocephalus-associated pain. Acetazolamide was included as a clinical reference compound in this context. Overall, these findings suggest that conventional analgesic therapies may provide some clinical benefit in managing pain in patients with hydrocephalus. Nevertheless, their limited and often suboptimal efficacy underscores the complexity of hydrocephalus-related pain and the pressing need for alternative strategies. Careful evaluation of the risk–benefit balance of current therapies is essential, as long-term use may be associated with several adverse effects or inadequate symptom control. Consequently, there is a strong and urgent demand for research focused on the development of safer, mechanism-based, and more precisely targeted treatments aimed at improving the quality of life of these patients.

After characterizing the sensory alterations through the behavioural test battery, and after establishing a time point -corresponding to the third week after kaolin injection (PND42, Fig. 3A)- that correspond to the time of maximal and stable ventricular enlargement [11] and that in our setting was free from significant changes in motor activity and resistance (Fig. 1H and I), we proceeded with histological analyses aimed at highlighting the key features of hydrocephalus pathology. We developed in our laboratory a macroscopic damage scoring system for the brains of animals with hydrocephalus, based on tissue appearance and structural integrity. Higher damage scores significantly correlated with increased brain water content, supporting the reliability of the scoring system (Fig. 3Ba-c). In our experimental setting, we confirmed the onset of ventricular enlargement -typical of hydrocephalus- through Evans index scores calculated on ex vivo sections [35]. In animals injected with kaolin, the indices are consistently higher compared to controls. The analysis then focused on the lateral ventricles, characterizing the immunofluorescence expression levels of GFAP, a glial marker used to visualize ependymal cells and astrocytes [36], and AQP4, a functional marker involved in the regulation of water exchange [37]. We observed that, at the level of the ependymal layer, both GFAP and AQP4 were overexpressed in animals with hydrocephalus (Fig. 3Da-d). This increase was also hinted at the surrounding circumventricular area. These findings may be interpreted as indicative of ependymal cell activation, potentially reflecting an enhanced transependymal flow response aimed at counteracting elevated intraventricular pressure [38, 39].

We then examined the choroid plexus in the lateral ventricles, due to its pivotal role in CSF homeostasis [40]. The choroid plexus exhibited the only significant histological sex-related difference, both in terms of fluorescent labelling for AQP1 -a channel protein involved in CSF secretion (Fig. 3Fa, Fb, Fi)- which was decreased in males and unchanged in females, and in terms of gene expression of other molecular effectors such as CAIII, CAIV, and GFAP (Fig. 3Fc-h), which were unchanged in males but modulated in females. Literature reports suggest that female hormonal fluctuations can affect choroid plexus function [41]. It would be worth investigating how this differential tissue response may impact the behavioural outcomes we observed, wherein females, three weeks post-injury, displayed lower scores in spontaneous pain assessments (Fig. 1Fb) and exhibited a robust anxiety-like behavioural profile (Fig. 1Gb), in contrast to males at the week 3/PND42 time point.

We then characterized the astrocytic profile in CNS regions involved in pain modulation, specifically: the somatosensory cortex, periaqueductal gray and dorsal horns of the spinal cord. The focus on astrocytes was based on a dual rationale. Astrocytes are key glial cells in regulation of synaptic transmission homeostasis through the coordinating action of AQP4. By facilitating water movement, AQP4 guides the osmotic gradients that enable the transport of ions and neurotransmitters [42]. In recent years, astrocytic AQP4 has gained attention as a potential modulator of pathological pain (reviewed in [43])*.* Moreover, due to the presence of AQP4, astrocytes also play a crucial role in CNS fluid dynamics by regulating exchanges between CSF in perivascular spaces and parenchymal interstitial fluid. This mechanism supports solute distribution, and clearance via diffusion process [44].

Analysis of the selected CNS regions revealed an area-dependent pattern of astrocytic alteration. Starting from the cerebral cortex, astrocyte changes appeared to be more qualitative than quantitative. While fluorescence intensity levels of the markers GFAP and AQP4 remained unchanged in this region (Fig. 4Ab-e), morphological examination revealed a marked simplification of astrocytic structure, with a reduction in both number and complexity of cellular processes (Fig. 4Af-i). This structural impoverishment may reflect diminished functionality, including impaired regulation of neural signalling and water homeostasis. The situation appeared even more compromised in the periaqueductal gray. Notably, consistent with observations in the ependymal layer of the lateral ventricles, we detected activation of ependymal cells lining the aqueduct (Fig. 4Bb and Bd), which may indicate local pressure imbalances. This alteration was accompanied by significant astrocytic distress in the surrounding parenchymal region, as evidenced by decreased fluorescence intensity of both GFAP and AQP4 (Fig. 4Cb-e), reduced density of GFAP-positive cells (Fig. 4Cf and Cg) and simplification of astrocytic morphology (Fig. 4Ch-m). In dorsal horns of the spinal cord we observed clear astrocytic activation, hallmark of neuropathic pain conditions and central sensitization [45], reflected by increased GFAP and AQP4 immunoreactivity (Fig. 4Db-e), a higher density of GFAP-positive cells (Fig. 4Df and Dg) and increased morphological complexity (Fig. 4Dh-Dm).

We propose that these two alterations could reflect distinct pathophysiological mechanisms operating at different levels of the neuraxis. The PAG is a multifunctional midbrain structure involved in the regulation of several physiological and pathological behaviours, including pain modulation, anxiety and defensive responses. With regard to pain specifically, the PAG exerts bidirectional control over nociception, being capable of both facilitating and suppressing pain processing depending on the subregion activated and on the behavioural, emotional and pathological context [46, 47]. The PAG is also anatomically adjacent to the cerebral aqueduct and therefore may be directly exposed to the mechanical stress imposed by elevated intraventricular pressure in hydrocephalus. The reduction in astroglial markers expression observed in this region could therefore be interpreted as reflecting primary astrocytic dysfunction driven by local compressive and hydrodynamic damage, a loss-of-function potentially consequent to direct cellular distress.

The alterations in the spinal dorsal horn, by contrast, being anatomically distant from the primary site of injury, may be considered as a secondary and distinct pathological mechanism. The upregulation of astroglial markers observed here would be more consistent with a reactive astrocytic response secondary to persistent and altered nociceptive input, potentially representing a hallmark of central sensitization in this persistent pain model.

The two alterations could reflect different pathophysiological processes: primary damage at the supraspinal level, and secondary reactive changes at the spinal level. The precise contribution of PAG dysfunction to spinal cord changes remain to be clarified, given the well-established complexity and multifunctionality of this region [46]. Nevertheless, the coexistence of astrocytic distress in the PAG with the behavioural profile of both cephalic and peripheral hypersensitivity and anxiety-like behaviours observed in our animals would suggest that this area plays a relevant role in the overall pathological picture of hydrocephalus-associated pain.

It is worth noting that GFAP has been increasingly recognized in the literature as a molecular biomarker of pain-related CNS pathology. In preclinical models, spinal astrogliosis (defined by upregulation of GFAP expression and morphological changes in astrocytes) has been consistently documented across a wide range of chronic pain models, including nerve trauma, spinal cord injury, chronic opioid exposure, bone cancer, chemotherapy-induced neuropathy, and HIV-induced neuropathy [7]. More recently, elevated serum GFAP levels have been reported in patients with chronic migraine compared to healthy controls, supporting its translational relevance as a biomarker of astrocytic damage in chronic pain conditions [48, 49].

Given that the PAG and the dorsal horns of the spinal cord emerged as the CNS regions most markedly affected by astrocytic alterations, we extended our analysis to microglia (Fig. 5), another key glial cell type critically involved in the mediation of central sensitization and neuroinflammatory states [8, 9]. Microglial cells were classified as resting or activated based on qualitative evaluation of their appearance: resting microglia displayed a ramified morphology with long, highly branched processes, while activated microglia were identified by cell body hypertrophy and marked reduction in process length and complexity. In both regions, kaolin-induced hydrocephalus was associated with a shift in microglial phenotype from a resting to an activated state, as evidenced by the increased percentage of morphologically activated Iba1-positive cells. In the dorsal horns of the spinal cord, this phenotypic shift was accompanied by a pronounced increase in the density of Iba1-positive cells, mirroring the proliferative astrocytic response observed in the same region and providing evidence of a neuroinflammatory profile, consistent with the hypothesized central sensitization. In the PAG, however, no significant change in microglial cell number was detected. As previously discussed in the context of astrocytic markers, this finding may be partly attributable to the mechanical compression exerted by aqueductal enlargement in this region, which could influence cell counts by inducing cellular distress.

### Limitations and future directions

While the region-specific pattern we document is consistent with existing literature on glial involvement in pain modulation, future mechanistic studies are needed to establish formal causality, such as localized drug delivery or gene editing, to dissect the contribution of individual brain areas to persistent pain. Immunofluorescence data, while revealing a clear and regionally consistent pattern of glial dysregulation, do not allow direct inference about the functional status of these cells. Altered marker expression need further validation to define a functional output, and this represents a recognized limitation of the descriptive approach here employed. Besides glial cells, the present study did not include neuronal markers or functional assessments of neuronal viability in the CNS regions of interests. Future studies incorporating neuronal markers and functional readouts will be essential to provide a more complete picture of the central and peripheral mechanisms underlying hydrocephalus-associated pain.

## Conclusion

In conclusion, this study highlights how the induction of hydrocephalus via kaolin injection in rats leads to a significant reduction in pain threshold, manifested by the onset of cephalic and peripheral thermal and mechanical hypersensitivity and spontaneous pain. This pain is of mixed origin, involving both nociceptive and neuropathic components, for which current gold-standard pain therapy offers only partial efficacy. At the histological level, we observed marked activation of ependymal cells, likely in response to increased intraventricular pressure, imposing mechanical and pressure-related stress on the surrounding tissue. In brain regions involved in pain modulation, such as the somatosensory cortex and periaqueductal gray, we documented signs of astrocytic distress, including reduced immunoreactivity, decreased astrocyte density and morphological complexity, and a drop in AQP4 levels. Moreover, the analysis of microglial cells in the periaqueductal gray revealed a shift towards an activated state, providing further evidence of cellular distress in this region. These alterations may reflect impairments in both synaptic regulation and hydrodynamic homeostasis. In contrast, the dorsal horns of the spinal cord exhibited increased GFAP and AQP4 fluorescence intensity and a higher density of GFAP-positive cells, indicating astrocyte activation and a central sensitization profile characteristic of persistent pain states. Consistently with this hypothesis, we observed a marked proliferation of microglial cells at the spinal level, together with an increased percentage of activated cells, supporting the idea a status of central sensitization and neuroinflammation. The region-specific heterogeneity in molecular alterations reveals a complex pathophysiological landscape, calling for mechanistic studies using precise modulation strategies, such as localized drug delivery or gene editing, to dissect the contribution of individual brain areas to persistent pain. The correlation we observed between impaired CSF homeostasis and reduced pain thresholds suggests that targeting CSF dysregulation could be a promising direction for developing pain-controlling strategies. Interventions aimed at restoring physiological CSF dynamics, such as modulation of aquaporins or approaches that reduce intracranial fluid accumulation, may offer therapeutic benefit and deserve further in-depth research. Such efforts are crucial to address an important clinical need that still remains unmet.

## Supplementary Information

Below is the link to the electronic supplementary material.

Supplementary Material 1

## Data Availability

The datasets used and/or analysed during the current study are available from the corresponding author on reasonable request.

## Abbreviations

AQP4: Aquaporin-4
CSF: Cerebrospinal fluid
CNS: Central nervous system
GFAP: Glial fibrillary acid protein
Iba1: Ionized calcium-binding adapter molecule 1
qPCR: Quantitative Real-Time PCR
