# Supplementary material for: Hydrocephalus-associated persistent pain: a preclinical investigation correlating brain fluid imbalance and sensory alterations
Source: Fluids Barriers CNS. 2026 Jul 7;23:116. doi: 10.1186/s12987-026-00838-x (PMC13629091; doi:10.1186/s12987-026-00838-x)
Supplement: Supplementary file 1 [file 12987_2026_838_MOESM1_ESM.docx]

**Supplementary Materials**

Supplementary figures and figure legends

**Supplementary Figure S1.** The time chart recapitulates the total number of male and female rats implied in each experimental setting. The actual data numerosity for each measurement is detailed in the figure legends.

**Supplementary Figure S2. Elevated plus maze test, plane distance travelled (m)**. male 1 week, ctr n = 6, kaol n = 7, **A**; female 1 week, ctr n = 7, kaol n = 8, **B**; male 3 weeks, ctr n = 9, kaol n = 7, **A**; female 3 weeks, p=0.0224, ctr n = 5, kaol n = 6, **B**; male 5 weeks, ctr n = 6, kaol n = 6, **A**; female 5 weeks, ctr n = 4, kaol n = 5, **B**.

**Supplementary Figure S3.** Representative EPM trajectory plots.

**Supplementary Figure S4.** **Time course evaluation of anxiety-like behaviour through elevated plus maze test.** For each time point, results related to entries, time and time mobile in centre, open arms and dark arms are displayed. Data are presented as mean ± SD. Statistical comparisons were performed using the two-sided Mann–Whitney test. Significance thresholds were set at p<0.05 (*) and p<0.01 (**). Numerosity: male 1 week, ctr n = 6, kaol n = 7, female 1 week, ctr n = 7, kaol n = 8; male 3 weeks, ctr n = 9, kaol n = 7, female 3 weeks, ctr n = 5, kaol n = 6 (centre entries p = 0.00811, centre time p = 0.01371, centre time mobile p = 0.0081, open arms entries p = 0.0215, open arms time p = 0.0357, open arms time mobile p = 0.0357, dark arms entries p = 0.0102, dark arms time p = 0.0224); male 5 weeks, ctr n = 6, kaol n = 6 (open arms entries p = 0.0136, open arms time p = 0.0139, open arms time mobile p = 0.0247, dark arms time p = 0.0306, open arms entries p = 0.0326, open arms time mobile p = 0.0373), female 5 weeks, ctr n = 4, kaol n = 5.

**Supplementary Figure S5.** Representative trajectory plots for Open Field.

**Supplementary Figure S6. Licking latency (s) recorded with Cold Plate test in the Brain Water Content animal cohort.** Licking latency in seconds measured with Cold Plate test in control animals and animals with kaolin-induced hydrocephalus, in male (**A**, p = 0.00729, ctr n = 10, kaol n = 6) and female (**B**, p = 0.00503, ctr n = 6, kaol n = 7).

**Supplementary Figure S7. Complete immunofluorescence data from brain ventricles.** Brain ventricles – ependyma. **A)** GFAP-positive area (%) in male rats (p=0.0142). **B)** GFAP-positive area (%) in female rats (p=0.0306). **C)** GFAP integrated density in male rats (p=0.0699). **D)** GFAP integrated density in female rats (p=0.00507). **E)** AQP4-positive area (%) in male rats (p=0.241). **F)** AQP4-positive area (%) in female rats (p=0.298). **G)** AQP4 integrated density in male rats (p=0.0699). **H)** AQP4 integrated density in female rats (p=0.00824). **I)** Field of view area (µm²) in male rats (p=0.110). **J)** Field of view area (µm²) in female rats (p=0.0453). Brain ventricles – circumventricular area. **K)** GFAP-positive area (%) in male rats (p=0.594). **L)** GFAP-positive area (%) in female rats (p=0.230). **M)** GFAP integrated density in male rats (p=0.0252). **N)** GFAP integrated density in female rats (p=0.173). **O)** AQP4-positive area (%) in male rats (p=0.749). **P)** AQP4-positive area (%) in female rats (p=0.575). **Q)** AQP4 integrated density in male rats (p=0.455). **R)** AQP4 integrated density in female rats (p=0.378). **S)** Field of view area (µm²) in male rats (p=0.337). **T)** Field of view area (µm²) in female rats (p=0.230). Numerosity of brain ventricles: male ctr n = 6, male kaol n = 4, female ctr n = 6, female kaol n = 6. Data are presented as mean ± SD. Statistical comparisons were performed using the two-sided Mann–Whitney test. First significance threshold was set at p < 0.05 (*). Second significance threshold was set at p < 0.01 (**).

**Supplementary Figure S8. Complete immunofluorescence data from choroid plexus**. **A)** AQP1-positive area (%) in male rats (p=0.315). **B)** AQP1-positive area (%) in female rats (p=0.575). **C)** AQP1 integrated density in male rats (p=0.927). **D)** AQP1 integrated density in female rats (p=0.379). **E)** Field of view area (µm²) in male rats (p=0.523). **F)** Field of view area (µm²) in female rats (p=0.936). Numerosity of choroid plexus: male ctr n = 6, male kaol n = 6, female ctr n = 6, female kaol n = 6. Data are presented as mean ± SD. Statistical comparisons were performed using the two-sided Mann–Whitney test.

**Supplementary Figure S9.** **Complete immunofluorescence data from somatosensory cortex**. **A)** GFAP-positive area (%) in male rats (p=0.915). **B)** GFAP-positive area (%) in female rats (p=0.936). **C)** GFAP integrated density in male rats (p=0.749). **D)** GFAP integrated density in female rats (p=0.298). **E)** AQP4-positive area (%) in male rats (p=0.915). **F)** AQP4-positive area (%) in female rats (p=0.689). **G)** AQP4 integrated density in male rats (p=0.594). **H)** AQP4 integrated density in female rats (p=0.230). **I)** Field of view area (µm²) in male rats (p=0.392). **J)** Field of view area (µm²) in female rats (p=0.0650). Numerosity of somatosensory cortex: male ctr n = 6, male kaol n = 4, female ctr n = 6, female kaol n = 6. Data are presented as mean ± SD. Statistical comparisons were performed using the two-sided Mann–Whitney test.

**Supplementary Figure S10. Complete immunofluorescence data from periaqueductal gray area.** Periaqueductal gray area – ependyma. **A)** GFAP-positive area (%) in male rats (p=0.648). **B)** GFAP-positive area (%) in female rats (p=0.246). **C)** GFAP integrated density in male rats (p=0.0358). **D)** GFAP integrated density in female rats (p=0.0282). **E)** AQP4-positive area (%) in male rats (p=0.784). **F)** AQP4-positive area (%) in female rats (p=1). **G)** AQP4 integrated density in male rats (p=0.784). **H)** AQP4 integrated density in female rats (p=0.519). **I)** Field of view area (µm²) in male rats (p=0.617). **J)** Field of view area (µm²) in female rats (p=0.366). Periaqueductal gray area – parenchyma. K) GFAP-positive area (%) in male rats (p=0.523). **L)** GFAP-positive area (%) in female rats (p=0.0282). **M)** GFAP integrated density in male rats (p=0.0552). **N)** GFAP integrated density in female rats (p=0.0282). **O)** AQP4-positive area (%) in male rats (p=0.784). **P)** AQP4-positive area (%) in female rats (p=0.898). **Q)** AQP4 integrated density in male rats (p=0.171). R) AQP4 integrated density in female rats (p=0.245). **S)** Field of view area (µm²) in male rats (p=0.787). **T)** Field of view area (µm²) in female rats (p=0.784). Numerosity of periaqueductal gray area: male ctr n = 6, male kaol n = 5, female ctr n = 6, female kaol n = 3. Data are presented as mean ± SD. Statistical comparisons were performed using the two-sided Mann–Whitney test. First significance threshold was set at p < 0.05 (*).

**Supplementary Figure S11. Complete immunofluorescence data from dorsal horns of the spinal cord.** **A)** GFAP-positive area (%) in male rats (p=0.00368). **B)** GFAP-positive area (%) in female rats (p=0.00811). **C)** GFAP integrated density in male rats (p=0.00241). **D)** GFAP integrated density in female rats (p=0.00811). **E)** AQP4-positive area (%) in male rats (p=0.478). **F)** AQP4-positive area (%) in female rats (p=0.784). **G)** AQP4 integrated density in male rats (p=0.0240). **H)** AQP4 integrated density in female rats (p=0.0358). **I)** Field of view area (µm²) in male rats (p=1). **J)** Field of view area (µm²) in female rats (p=1). Numerosity of dorsal horns of the spinal cord: male ctr n = 6, male kaol n = 8, female ctr n = 5, female kaol n = 6. Data are presented as mean ± SD. Statistical comparisons were performed using the two-sided Mann–Whitney test. First significance threshold was set at p < 0.05 (*). Second significance threshold was set at p < 0.01 (**).
